# The association between polyomavirus BK strains and BKV viruria in liver transplant recipients

**DOI:** 10.1038/srep28491

**Published:** 2016-06-24

**Authors:** Robert Y. L. Wang, Yi-Jung Li, Wei-Chen Lee, Hsin-Hsu Wu, Chan-Yu Lin, Cheng-Chia Lee, Yung-Chang Chen, Cheng-Chieh Hung, Chih-Wei Yang, Ya-Chung Tian

**Affiliations:** 1 Department of Biomedical Sciences, College of Medicine, Chang Gung University, Taoyuan, Taiwan; 2 Molecular Medicine Research Center, Chang Gung University, Taoyuan, Taiwan; 3 Kidney Research Center and Department of Nephrology, Chang Gung Memorial Hospital, Taipei, Taiwan; 4 Department of Medicine, Chang Gung University, Taoyuan, Taiwan; 5 Graduate Institute of Clinical Medical Sciences, Chang Gung University, Taoyuan, Taiwan; 6 Department of General Surgery, Chang Gung Memorial Hospital, Taipei, Taiwan

## Abstract

BK virus (BKV) is a polyomavirus that cause of allograft dysfunction among kidney transplant recipients. The role of BKV infection in non-renal solid organ transplant recipients is not well understood neither for the relationship between various BKV strains with occurrence of BKV viral viruria. This study aimed to understand the prevalence of BKV infection and identified of BKV various strains in the urine of liver transplant recipients. There was not significant difference of renal outcome between high BKV viruria and low BKV viruria in the liver transplant recipients. The WW-non-coding control region (NCCR) BKV detected in urine was associated with higher urinary BKV load, whereas the Dunlop-NCCR BKV was detected in the urine of low urinary BKV load. An *in vitro* cultivation system demonstrated that WW-BKV strain exhibiting the higher viral DNA replication efficiency and higher BKV load. Altogether, this is the first study to demonstrate the impact of BKV strains on the occurrence of BK viruria in the liver transplant recipients.

Polyomavirus BK (BKV) infection has emerged as a serious complication that affects renal function in transplant recipients. It has been well documented that BKV infection can cause polyomavirus-associated nephropathy (PVAN) and leads to allograft loss in renal transplant patients^1^,^2^,^3^,^4^. Whether BKV infection in liver transplant patient results in the deterioration of renal function is still remains controversial. Loeches *et al*. observed in a prospective study that persistent BKV viremia might be related to the impaired renal function in liver transplant recipients^5^. In contrast, other studies did not discover the association between BKV viremia and viruria with renal dysfunction in liver transplant patients^6^,^7^,^8^. The discrepancy in high association of BKV infection with renal allograft function in renal transplant recipients and no obviously causal relationship between BKV infection and renal function in liver transplant recipients may highlight the importance of the second hit such as ischemia or inflammation in the development of renal dysfunction in addition to BKV infection.

BKV reactivation is early discovered in the shed and infected urothelial cells. BKV viruria can be frequently detected prior to the occurrence of BKV viremia and the development of PVAN and indicates rapid replication of virus in the urothelial cells has been reported in 10–60% of renal transplant recipients^9^,^10^,^11^,^12^,^13^. BKV reactivation is often caused by failure of immune surveillance due to the use of high-dose immunosuppressants and aggressive viral replication that depends on viral strains and the host microenvironment.

The various strains of BKV exhibit a highly possibility of heterogeneity in the transcriptional control region, is believed to affect the replicating efficiency of BKV in host. It is known that the BKV viral genome is divided into two protein-coding regions that are transcribed in opposite directionsstarting from a common noncoding control region (NCCR). The NCCR constitutes a regulatory region, which is bidirection, that it contains promoter/enhancer elements for the viral early and late genes and also contains the origin of viral DNA replication.

The BKV NCCR has been well characterized by the highly degree of variation between strains due to the occurrence of multiple rearrangements in the later proximal enhancer element observed from different isolates, which were obtained from cell cultures and patients’ specimen. Two major types of BKV NCCR: WW strain (GenBank accession no. M15987), the archetypal or unrearranged NCCR, is predominant in the urine and is the transmissible form of the virus^14^,^15^. The NCCR of WW strain is arbitrarily divided into five regions, named the O, P, Q, R, and S elements^14^,^16^,^17^. The O element is the early proximal element between the start codon of T-antigen and the 5′ end of the enhancer element, including the origin of DNA replication, followed by the other elements P, Q, R, and S. S element is the leader region of the late transcription; Dunlop strain (GenBank accession no. V0ll08), the prototypical NCCR, has a rearranged NCCR with the configuration OPP’P”S, i.e., the late proximal control region contains an imperfect triple-repeat enhancer element^14^. The BKV NCCR early promoter activity is dependent on elements that are located on both upstream and downstream of the transcription start site^18^.

Several factors such as new-onset diabetes, underlying causes of liver failure and nephrotoxicity of immunosuppressants may contribute to renal dysfunction in liver transplant recipients^19^,^20^,^21^. There are some studies showed that BKV prevalence plays roles in renal dysfunction in many solid organ transplants with documented cases of BKV nephropathy after receiving heart, lung, and pancreas transplantation. In the case of liver transplant recipients, *Munoz et al.,* reported that the prevalence of BKV viremia was between 0–2.8% and the prevalence of viruria was between 8–16% with a 4.1% first-year incidence in 121 enrollments. However, the clinical relevance of BKV infection in the liver transplant recipients has not well understood. The aim of the current study is to investigate the prevalence of BKV viruria in liver transplant recipients and to clarify whether the various BKV strains are associated with the occurrence of BKV viruria in the liver transplant recipients.

## Results

### Baseline demographic characteristics

Twenty-two liver transplant recipients received screening for urinary BKV load since March 2009 and were prospectively followed up to October 2013. Urine samples were collected within three months after liver transplantation. The recipients were divided into two groups according to urinary BKV load. The high BKV viruria (high urinary BKV load) group (9 subjects) was defined as urinary BKV copy number in any urine sample was more than 50 × 10^3^ copies/mL and the rest (13 subjects) was enrolled into the low BKV viruria (low urinary BKV load) group. This classification was based on our previous observation that kidney transplant recipients with urinary BKV load more than 50 × 10^3^ copies/mL were more likely to have renal function decline during follow-up^3^. Demographic data demonstrated that the recipients in the high BKV viruria group were older than those in the low BKV viruria group. There was no difference in sex, the presence of diabetes, the donor type (living-related or cadaveric), HBV infection and HCV infection in these two groups (Table 1). The use of immunosuppressants including tacrolimus, mycophenolate and steroids in both groups were similar. Hemogram demonstrated no significant difference in hematocrit and the number of platelets in two groups but a higher WBC count in the low BKV viruria group. Clinical biochemistry data showed a comparable serum level of albumin, bilirubin, AST and ALT in both groups. The proportion of patients with proteinuria was not different between both groups.

### Comparison of the outcomes in liver transplant recipients with high or low BKVviruria

**Figure 1:**
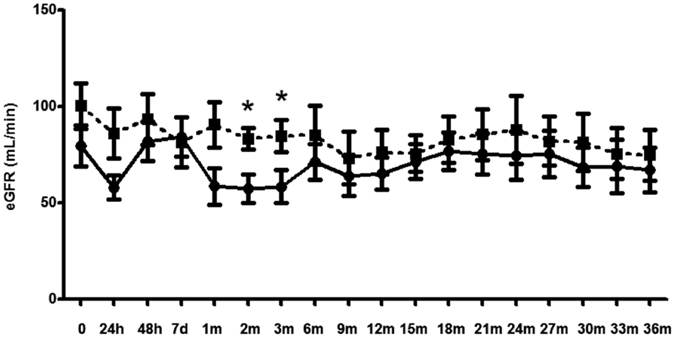
Comparison of the eGFR value between the low BKV viruria and high BKV viruria groups. The eGFR measured by Cockcroft-Gault formula in the low (solid line) and high (dash line) BKV viruria groups was assessed on the operation day, 24 h, 48 h, 7 d, one month, two months, three months and then every 3 months after the surgery. The star sign *indicates the significant difference in eGFR value in these two groups (*p* < 0.05).

To assess the renal outcome in liver transplant patients between high and low BKV viruria, eGFR was examined on the operation day, 24 h, 48 h, 7 days, one month, two months, three months and followed up every 3 months after the surgery for 36 months. The results demonstrated that eGFR was not significantly different in both groups except for the 2^nd^ and 3^rd^ months (Fig. 1). Five recipients (39%) in the low BKV viruria group and 3 patients in the high BKV viruria group (33%) required hemodialysis during the follow-up period. Five patients in the low BKV viruria group and 3 patients in the high BKV viruria group died due to the liver allograft failure complicated with sepsis during 36-month follow-up. The requirement of hemodialysis and the mortality rate were not significantly difference between both groups during the follow-up.

### The association of the BKV strains with urinary BKV load

**Figure 2:**
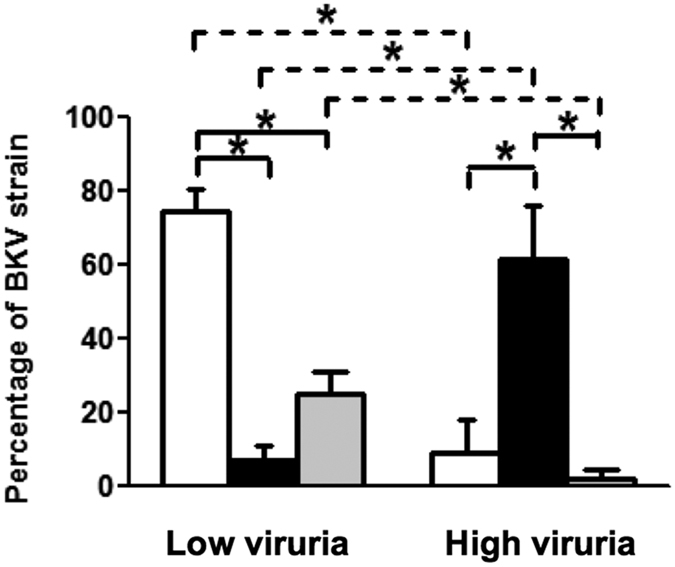
Analysis of major BKV strains in low and high BKV viruria urine samples BKV strains were determined in forty-three urine samples from 22 liver transplant recipients. BKV load was more than 5 × 10^4^ copies/mL in 11 urine samples (high urinary BKV load; high viruria) and less than 5 × 10^4^ BKV copies/mL in 32 urine samples (low urinary BKV load; low viruria). NCCR amplification used a standard nested PCR and gene cloning and sequencing technique to analyze BKV strains as described in the Materials and Methods section. The percentages of two major strains (Dunlop strain: white bar; WW strain: black bar) and other strains categorized into one group (grey bar) in low and high viruria samples were displayed respectively. Star sign * indicates significant difference in comparisons (*p* < 0.01).

To determine the association between BKV strains and the urinary BKV load, the NCCR architecture was analyzed from the sequenced of PCR product and classified in different strains in according to the description as shown in the Materials and Methods. We sequenced NCCR in 43 urine samples from 22 liver transplant recipients as described in the Materials and Methods section. In these 43 urine samples, BKV load was more than 50 × 10^3^ copies/mL in 11 urine samples (high urinary BKV load) and the other 32 urine samples contained less than 50 × 10^3^ BKV copies/mL (low urinary BKV load) (For the summarizing the urine sampling results, please see the Supplementary Table S1). The percentages of BKV strains in low and high BKV load urine samples were shown in Fig. 2. The Dunlop strain was the most commonly detected in the low urinary BKV load samples , while the WW strain was the major strain detected in the high urinary BKV load samples.

To further analyze the association between urinary viral load and the major strains, BKV titer in the urine samples with the Dunlop or WW strain as the dominant strain defined as the discovered frequency of one specific strain was more than 50% in one urine sample was calculated. In accordance with above findings, the average of the BKV titer in the WW strain-dominant urine samples was 210 × 10^3^ copies/mL, while that in the Dunlop strain-dominant urine samples was only 15 × 10^3^ copies/mL (*p* < 0.05). In fact, BKV loads in 27 urine sample out of the 28 Dunlop strain-dominant urine samples were less than 50 × 10^3^ copies/mL and only one urine sample had BKV load more than 50 × 10^3^ copies/mL (75 × 10^3^ BKV copies/mL). Five out of the 7 WW strain-dominant urine samples had BKV load more than 50 × 10^3^ copies/mL. The positive predict value of the Dunlop strain dominance in predicting low BKV viruria was 0.96 and that of the WW strain dominance in predicting high BKV viruria was 0.71.

### WW- and WW-like-NCCR increase BKV replication *in vitro*

**Figure 3:**
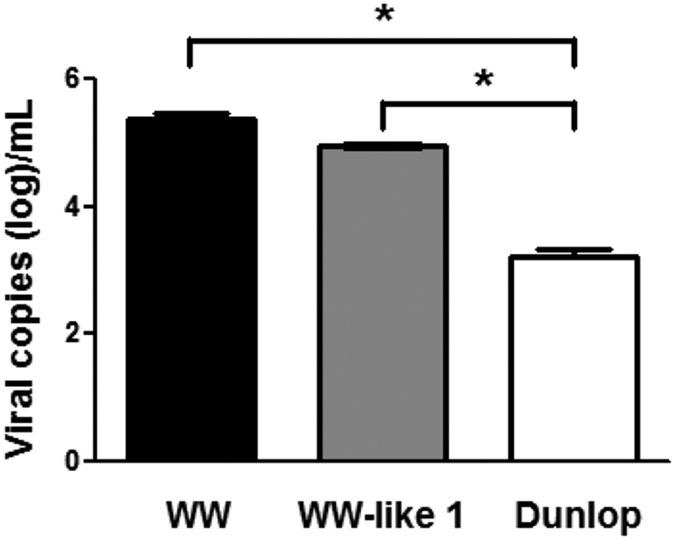
BKV WW strain replicates faster than BKV Dunlop and WW-like 1 strain in cell culture. Three recombinant viruses were generated using the Dunlop-BKV backbone and NCCR of the WW or WW-like-1 strains isolated from the urine of liver transplant recipients. BKV DNA loads in the cell lysates of transfected HK2 cells were measured by quantitative PCR. Data are representative of three independent experiments. (*indicates *p* < 0.01).

Since it has been reported that significant sequence variability in the NCCR of BKV isolates from different human sources, it is believed that unique DNA sequence variations in this region (but not found in any other region of the BKV full DNA genome) playing a role in the pathogenesis of PVAN. We have successfully propagated three BKV strains (Dunlop-BKV, WW-BKV and WW-like 1-BKV; unpublished data) in the human kidney epithelial cell line HK-2. Hence, we employed this *in vitro* cultivation system to examine the viral replication efficiency among different BKV strains. Three BKV strains infection were initiated by Lipofectamin (Life technology) transfection of 75% confluent monolayers of HK2 cells, with circularized Dunlop-, WW-, and WW-like 1-BKV genomes. On beforehand the complete BKV DNA genomes were cut from pBR322-BKVs using *Bam H1* and re-ligated. The presence of viral DNA in culture supernatant was determined at 3 dpt (days post-transfection). As a result, viral copies in the supernatant of the WW-, WW-like 1-and Dunlop-BKV transfected cells were 244.2 × 10^3^ (5.37 log), 87.6 × 10^3^ (4.94 log) and 1.7 × 10^3^ (3.22 log), respectively. There was a 144-fold increase amount of viral DNA detected from the WW-BKV transfectant cells in comparison with the Dunlop-BKV transfectant cells. Similarly, there was also a 52-fold increase of viral DNA detected from the WW-like 1-BKV transfectant cells (Fig. 3), indicating the WW strain facilitated the higher viral DNA replication efficiency.

## Discussion

Many factors affect viral replication, including the virulence of different viral strains, local environment and the immune status in the host. Following primary infection of BKV, BKV remain latent in the urorenal tract and most of infected individuals are symptomless throughout the life. The hypothesis of the second hit due to kidney tissue damage caused by surgery, rejection or immunosuppressive agents have been proposed to highlight the importance of tissue injury in the development of PVAN^22^. Since kidney tissue damage caused by ischemia and inflammation in renal transplant recipients may affect urinary BKV load, the current study enrolled the liver transplant recipients instead of kidney transplant recipients to assess the association between BKV strains and urinary BKV load, thus reducing the influence of kidney tissue damage on the alteration of urinary BKV load.

It has been reported that the prevalence of BKV viruria in liver transplant recipients is 8–21%, depending on the definition of BKV viruria and detection methods^5^,^6^,^23^,^24^. In this study, we demonstrated that 9 patients of a total 22 patients (40%) had high BKV viruria during the first three months after liver transplantation. Higher prevalence of BKV viruria in this study compared with that reported in other studies may be attributed to higher dosages of immunosuppressants used in the first three months after liver transplantation, leading to more intensive immunosuppression in our subjects. Despite high prevalence of BKV viruria in liver transplant recipients, the renal outcome seems unassociated with the presence of BKV viruria^5^,^8^,^25^. Similarly, our data also demonstrated that eGFR was not significantly different in the high and low urinary BKV load groups except for the 2^nd^ and 3^rd^ months. We speculate that high urinary BKV load in the first three months after liver transplantation caused by the use of higher immunosuppressive therapy does not lead to significant kidney damage as long as ischemic or inflammatory kidney injury is not severe. In contrast, high urinary BKV load may superimpose kidney damage in transplant kidneys and is associated with renal function decline in kidney transplant recipients.

Since 1990 s, Sundsfjord *et al*. reported that BKV strains with two different types of NCCR were found in the urines of patients from Norway, the patients excreted BKV with various NCCRs were characterized by the highest degree of variation among strains were demonstrated as the occurrence of multiple rearrangements in the late proximal enhancer element observed for different clinical isolates^18^,^26^. Although high urinary BKV load is an important risk factor of developing PVAN, the association between the BKV strains and urinary BKV loads in these studies remains unclear. In this study, we demonstrated a strong association of urinary BKV load and BKV strains, as the WW strain was the most abundant strain isolated from urine samples with high urinary BKV load, while the Dunlop strain was the predominant strain in the urine samples with low urinary BKV load. The association between low urinary BKV load and the Dunlop strain dominance is strong as the positive predict value is 0.96. The association between high urinary BKV load and the WW strain dominance is less strong but the positive predict value is still up to 0.71. In line with the clinical findings, the results of BKV replication in the cell culture system also revealed that more viral DNA was detected following transfection with the WW strain plasmid in comparison with the Dunlop strain plasmid. This result indicates that the WW-NCCR facilitates the higher viral DNA replication efficiency and may reflect the clinical finding that the WW strain is the predominant strain detected from urine samples with high BKV load in liver transplant recipients. On NCCR, there are the binding sites of several transcription factors that have been identified to regulate viral replication^27^,^28^,^29^. For example, nuclear factor of activated T cells (NFAT) bound to the NCCR of BKV is essential for viral replication^30^,^31^. It would be interesting to elucidate whether the alterations in the binding sites of various transcription factors on NCCR causes the differences in the occurrence of BKV viruria and viral replication. Nevertheless, our results suggest that WW-NCCR in comparison of Dunlop-NCCR is sufficient to confer rapidly increased early gene expression and replication in the liver transplant recipients. At present, it is still unknown whether these various BKV strains existed in the original wild-type BKV strain or have accumulated and circulated during transmission in human host. Our results suggest that anatomy of the NCCRs may strongly influence the biological characteristics of a BKV strain with regard to host cell permissively and even the transforming abilities. In renal transplant recipients that have more severe renal ischemia and inflammation compared with liver transplant recipients, whether specific BKV strains are strongly associated with high urinary BKV load or the development of PVAN requires further elucidation.

The limitation of this study is a relatively small sample-sized study and only 43 urine samples were analyzed. However, although the renal outcome may require a larger study to verify the results, the association between BKV strains and urinary BKV loads in this study is clear, providing evidence that the Dunlop strain replicates slowly and is dominant in low urinary BKV load samples. Another limitation of this study is that other BKV strains with very low titer in urine samples cannot be detected using PCR and cloning techniques. Nevertheless, this drawback does not influence the association between the dominant strain and urinary BKV load.

In summary, we documented a clearly impact of different BKV NCCR variants contributing to high urinary BKV load early after liver transplantation. This is the first prevalence study of BKV viruria immediately following liver transplantation. A close relationship between BKV NCCR variants detected in urine and urinary BKV load can be well documented. *In vitro* study also verified the importance of BKV strains in viral DNA replication. Further large sample-size studies are needed to assess the long-term risks of BKV viruria in liver transplant recipients and allow us to identify the specific BKV strain in which increases the risk for the development of PVAN in different organ transplant recipients.

## Methods

All of the clinical specimens experimental protocols were approved by the Research Ethics Board of Chang Gung Memorial Hospital.

### Subjects and clinical specimens

This study enrolled 22 subjects who received liver transplantation in our center during May 2009 and September 2010 and were prospectively followed up after operation. Urine samples were collected from liver transplant patients in accordance with the guidelines approved by the institutional review board of Chang Gung Memorial Hospital in 2009. Urine samples were collected within three months post-liver transplantation after the informed consent was obtained. The informed consent was obtained from all enrolled subjects. We enrolled liver transplant recipients randomly and excluded those on dialysis due to no available urine samples and those who did not sign the informed consent. Demographic data, including age, gender, donor type (cadaveric or living donors), diabetes, the presence of hepatitis B virus (HBV) or hepatitis C virus (HCV) infection, hemogram, biochemistry of liver function and proteinuria were recorded. Estimated glomerular filtration rate (eGFR) using Cockcroft-Gault formula was determined on the operation day, and post-operation 24 h, 48 h, 7 d, 30 d, 60 d, and then measured every 3 months. The requirement of dialysis and patient’s death were recorded. Proteinuria was measured by Dipstick method and proteinuria more than 25 mg/dl was considered as positive.

### BKV load and NCCR analysis

Forty-three urine samples from 22 liver transplant recipients were collected for analysis of BKV strains. All of these recipients except one recipient who had only one urine sample had two urine samples collected for strain analysis. Urine BKV DNA was quantified after DNA extraction from 200 μl of urine using a commercial kit (QIAamp^®^ DNA Mini Kit, Qiagen, Hilden, Germany) and a previously described real-time PCR protocol^32^,^33^. In brief, a total volume of 50 μL PCR mixture, containing 900 nM of each primer, 100 nM of TagMan FAM-MGB probe, and 25 μL of the 2X TagMan Universal PCR Master Mix (Applied Biosystems). Real-time PCR was performed on an ABI**-**Prism 7700 using SYBR Green I as a double-stranded DNA-specific dye according to the manufacturer’s instructions (PE-Applied Biosystems, Cheshire, Great Britain). Glyceraldehyde-3-phosphate dehydrogenase (GAPDH) was used as a standard housekeeping gene. Primers (BKV: sense 5-CTGTCCCTAAAACCCTGCAA-3 and anti-sense 5-GCCTTTCCTTCCATTCAACA-3) were constructed to be compatible with a single RT-PCR thermal profile (95 °C for 10 min, 40 cycles of 95 °C for 30 s and 60 °C for 1 min). Accumulation of the PCR product was monitored in real time (PE-Applied Biosystems). The copy numbers of BKV were calculated based on the standard curve as described below. The assay showed a wide linear range from 10 to 10^9^ copy equivalents of viral DNA and the limit of detection was 10 copies/mL ofBKV DNA. NCCR amplification used a standard nested PCR approach with the inner primer pairs: primer #92 5′-GCAAAAATTGCAAAAGAATAGGGATTTCCCC-3′ and primer #93 5′-GTTCCAGTCCAGGTTTTACCAAC-3′ and the outer primer pairs: primer #206 5′-CACCCTTACTACTCGAGAGAAAGGGTGG-3′ and primer #145 5′-GCGACTAGTGGATCCCCCATTTCTGGGTTTAG-3′. The PCR products were then analyzed and compared after separation with 2.5% TAE-agarose gel electrophoresis with WW-NCCR control, followed by excising the indicated band for sequence analysis. Dilution series of increasing ratios of WW-like NCCR versus WW-NCCR and cloning into plasmids indicated that the sensitivity of detecting minority species in NCCR mixtures by the PCR protocol was as low as 3.5% (data not shown). Each colony containing one single NCCR was further subjected to gene sequencing for determination of BKV strain. The percentage of BKV strains in each urine sample was then calculated.

The fragment of BKV large T-antigen was amplified from BKV obtained from American Type Culture Collection (CCL-137, Manassas) and then cloned into the TOPO TA^®^ Cloning vector (Invitrogen, California USA). The plasmid containing a BK viral fragment was amplified and purifiedusing Genopure Plasmid Midi Kit (Roche, Mannheim Germany). Plasmid DNA concentrations were determined by OD measurement at 260nm. The copy numbers of BKV plasmids were calculated based on the following formula: *m* = *n* × *1.096* × *10*^*−21*^ *g/bp*; *M* = *m* × *copy number of interest* where *n* was the plasmid size (bp), *m* was the mass of DNA, and *M* was the mass required for obtaining the copy number of interest. The BKV plasmid DNA was serially diluted in a logarithmic manner to obtain 10^7^∼10^10^ copies per sample load (5 μl) for standard curve establishment. The correlation value of the standard curve was limited to *0.995* < *r*^*2*^ < *1*.

### Statistical analysis

All the data were presented as means ± standard deviation except means ± standard errors. All variables were tested for normal distributions using the Kolmogorov-Smirnov test. The Student’s *t-*test was applied to compare the means of continuous variables and normally distributed data; otherwise, the Mann-Whitney *U* test was employed. The difference of the categorical variances was analyzed by Pearson’s chi square test or Fisher’s exact test. A value of p < 0.05 was considered to represent a significant difference.

## Additional Information

**How to cite this article**: Wang, R. Y. L. *et al*. The association between polyomavirus BK strains and BKV viruria in liver transplant recipients. *Sci. Rep.* **6**, 28491; doi: 10.1038/srep28491 (2016).

## Supplementary Material

Supplementary Information

## Figures and Tables

**Table 1:** Demographic and laboratory characteristics.

	Total	Low viuria	High viruria	P-value
Sex (Male)	17 (77%)	12 (92%)	5 (56%)	0.116
Age	54.7 ± 9.4	51.5 ± 9.2	59.3 ± 7.8	0.048
DM	9 (41%)	6 (46%)	3 (33%)	0.674
Living donor	13 (59%)	6 (46%)	7 (78%)	0.203
HBV infection	10 (46%)	6 (46%)	4 (44%)	1.000
HCV infection	9 (41%)	5 (39%)	4 (44%)	1.000
Use of immunosuppressants				
Tacrolimus	20 (91%)	13 (100%)	7 (78%)	0.156
Mycophenolate	17 (77%)	12 (92%)	5 (56%)	0.116
Steroids	18 (82%)	11 (85%)	7 (78%)	1.000
Hemogram				
Hematocrit (%)	29.6 ± 4.8	29.7 ± 5.0	29.3 ± 4.8	0.332
WBC count (μL)	8257 ± 3789	9515 ± 4203	6213 ± 1734	0.012
Platelet count (1000/μL)	75.6 ± 46.5	75.5 ± 50.3	75.8 ± 42.7	0.813
Biochemistry				
Albumin (g/dL)	2.8 ± 0.5	2.9 ± 0.5	2.6 ± 0.7	0.245
Bilirubin (mg/dL)	6.1 ± 8.6	8.2 ± 10.4	2.7 ± 2.0	0.103
AST (U/L)	137 ± 261	166 ± 330	89 ± 59	0.414
ALT (U/L)	76 ± 112	88 ± 141	58 ± 29	0.455
Presence of proteinuria	8 (36%)	6 (46%)	2 (22%)	0.380
Requirement of dialysis	8 (36%)	5 (39%)	3 (33%)	1.000
Mortality	8 (36%)	5 (39%)	3 (33%)	1.000
