# Supplementary material for: The association between polyomavirus BK strains and BKV viruria in liver transplant recipients
Source: Sci Rep. 2016 Jun 24;6:28491. doi: 10.1038/srep28491 (PMC4919687; doi:10.1038/srep28491)
Supplement: Supplementary Information [file srep28491-s1.pdf]

Supplementary information:

Title:

The association between polyomavirus BK strains and BKV viruria in liver transplant recipients

Robert YL Wang, Yi-Jung Li, Wei-Chen Lee, Hsin-Hsu Wu, Chan-Yu Lin, Cheng-Chia Lee, Yung-Chang Chen, Cheng-Chieh Hung, Chih-Wei Yang, Ya-Chung Tian

Table S1

Table S1. List of urinary BKV load and multiple NCCR variants identified in the urine from liver transplantation patients

Patients ID								
	**Urine BKV copy number (DNA/mL)**	**NCCR strains**			**Urine BKV copy number (DNA/mL)**	**NCCR strains**		
		Dunlop	ww	ww-like		Dunlop	ww	ww-like
ID1	138,739	0	100	0	359,198	0	100	0
ID2	233,973	0	100	0	21,991	75	0	25
ID3	75,501	0	100	0	309,431	100	0	0
ID4	351,881	0	75	25	41,174	100	0	0
ID5	7,269	100	0	0	51,474	0	100	0
ID6	338	100	0	0	75,704	0	100	0
ID7	4006	75	0	25	351,881	0	100	0
ID8	415	100	0	0	278,769	0	100	0
ID9	363	0	0	100	672	72	0	28
ID10	62	100	0	0	62	0	20	80
ID11	1,203	67	0	33	833	0	0	100
ID12	1,209	0	0	100	924	100	0	0
ID13	3,511	100	0	0	1,353	90	0	10
ID14	325	0	0	100	1,140	80	0	20
ID15	1,417	100	0	0	10,512	72	0	27
ID16	1,393	0	100	0	1,393	75	0	25
ID17	362	100	0	0	125	100	0	0
ID18	371	100	0	0	366	100	0	0
ID19	3,869	78	0	22	1,768	100	0	0
ID20	1,222	100	0	0	1,707	100	0	0
ID21	12,931	67	0	33	10,984	75	0	25
